# The relationship between emotional intelligence and quality of life in schizophrenia and bipolar I disorder

**DOI:** 10.1007/s11136-021-02843-z

**Published:** 2021-05-05

**Authors:** Beatrice Frajo-Apor, Silvia Pardeller, Georg Kemmler, Moritz Mühlbacher, Anna-Sophia Welte, Christine Hörtnagl, Birgit Derntl, Alex Hofer

**Affiliations:** 1 Department of Psychiatry, Psychotherapy and Psychosomatics, Division of Psychiatry I, Medical University Innsbruck, Anichstr. 35, 6020 Innsbruck, Austria; 2 Department of Psychiatry and Psychotherapy, Paracelsus Private Medical University, Ignaz-Harrer-Straße 79, 5020 Salzburg, Austria; 3 Department of Psychiatry and Psychotherapy, University of Tübingen, 72076 Tübingen, Germany

**Keywords:** Quality of life, Emotional Intelligence, Schizophrenia, Bipolar disorder

## Abstract

**Purpose:**

Social cognitive skills, both psychosocial functioning and well-being of patients with schizophrenia (SZ) or bipolar disorder (BD), have consistently been shown to be interrelated. While previous research mainly focused on emotion perception, the present study investigates the impact of the other subdomains of emotion processing on a subjective Quality of Life (QoL) estimate and objective QoL indicators. We hypothesized that patients with better performance in the Mayer-Salovey-Caruso Emotional Intelligence Test (MSCEIT) report better QoL; and assumed that SZ and BD patients report comparable subjective QoL, whereas BD patients show higher levels of objective QoL.

**Methods:**

Patients diagnosed with either SZ (*n* = 63) or BD (*n* = 60), as well as 80 healthy controls, were included into a cross-sectional study. Emotional Intelligence (EI) and QoL were assessed using the MSCEIT and the German version of the Lancashire Quality of Life Profile.

**Results:**

The two patient groups were comparable with regard to overall EI, as well as subjective and objective QoL, but indicated significantly lower levels of EI and QoL than healthy controls. Whereas EI was not associated with both patient groups’ subjective QoL, a significant correlation of EI with objective QoL was only observed in SZ. However, overall effect sizes were small.

**Conclusion:**

Our findings point to a difference in the interrelation between EI and QoL in patients suffering from SZ and BD, and suggest that they may have different needs to achieve recovery. It will be critical to develop training programs targeting EI in SZ, and to examine their impact on objective QoL in these patients.

## Introduction

According to the World Health Organization (WHO), the term Quality of life (QoL) refers to “*an individuals’ perceptions of their position in life in the context of the culture and value systems in which they live, and in relation to their goals, expectations, standards and concerns. It is a broad ranging concept affected in a complex way by the persons’ physical health, psychological state, level of independence, social relationships and their relationship to salient features of their environment” *[1]*.*

Over the last decades, QoL has gained importance as an outcome measure in health research, and successful treatment nowadays not only describes a reduction of specific symptoms, but also aims at a holistic approach to reach recovery [2]. Therefore, QoL may not only be seen as a reliable indicator of disease burden [3], but is also an important treatment and research target, e.g. in patients suffering from serious mental illness (SMI), which is defined as “*mental, behavioral, or emotional disorder resulting in serious functional impairment, which substantially interferes with or limits one or more major life activities.” *[4]*.*

Patients suffering from SMI such as schizophrenia (SZ) or bipolar disorder (BD) report drastic impairments in QoL compared to healthy individuals [5, 6]. Schizophrenia is characterized by episodes of psychosis (delusions, hallucinations, disorganized thinking), as well as many other symptoms, such as cognitive deficits, social withdrawal, reduced emotional expression, or apathy. The course of the illness and the severity of symptoms can be very different from one individual to the other. Symptoms may occur continuously or as relapsing episodes. Bipolar disorder, on the other hand, is a mood disorder, mainly characterized by episodes of depressed or elevated mood. However, cognitive and psychotic symptoms may occur in this disorder as well, and both disorders are associated with high rates of suicidality and disability [7].

Cross-diagnostic comparisons have shown that patients with BD have better functional outcomes compared to those suffering from schizophrenia, but the two groups seem to achieve a comparable QoL in the course of illness [8–10]. A better understanding of the factors influencing QoL in SMI may help to develop targeted interventions to improve subjective recovery in those affected. Among others, depressive symptoms, medical and psychiatric comorbidities, and treatment adverse effects have consistently been shown to negatively predict QoL in SMI in general, while objective recovery, i.e. symptom remission, resilience, and social cognitive skills have been associated with better QoL [8, 11–13].

Generally, the term “social cognition” covers a broad field and comprises four primary domains: emotion processing, social perception, Theory of Mind (ToM) / mental state attribution, and attributional style / bias [14]. Previous research has documented the relationship between emotion processing abilities and QoL in BD. For example, deficits in recognizing fear [15] and in experiencing emotions have been demonstrated to be associated with reduced QoL in these patients [16]. In SZ, a number of studies have shown that social cognitive impairments exert a major influence on a patient’s psychosocial functioning and well-being [17–19]. While emotion processing has been shown to predict successful employment and independent living [20, 21], the correct recognition of facial affect and affective prosody has been associated with interpersonal relationship patterns [22], communication [23, 24], and occupational functioning [24]. The investigation of hospitalized SZ patients has yielded a positive association between facial affect recognition and social competence, social interests, and personal hygiene [25]. Notably, further investigation is needed to understand the interrelations between the other subdomains of emotion processing (using, understanding, and managing emotions) and QoL, as subjective elements of recovery. Additionally, the impact of the nature of SMI in this regard are still not well understood and should be examined. We therefore chose a comparative approach to identify cross-diagnostic differences between SZ and BD. Accordingly, the current study concentrates on the relationship between QoL and Emotional Intelligence (EI).

Emotional Intelligence, a concept consolidated by Salovey and Mayer [26], focuses on personality traits and abilities enabling people to cope with both their own and others’ feelings [27]. According to their “ability” model, EI is understood as a combination of emotion-specific abilities: perceiving, using, understanding, and managing emotions. “Perceiving emotions” means the ability to recognize emotions accurately, while “using emotions” is about using emotions to enhance cognitive processes. “Understanding emotions” is the knowledge of how emotions interact with each other and change over time, and “managing emotions” means the ability to deal with and regulate emotions. This model suggests that EI is a skill, which can be developed and trained, in order to improve social cognitive abilities. Moreover, it can be measured objectively along the lines of conventional intelligence tests, which was the main reason for choosing it for the present investigation. In contrast, the “trait model” of Emotional Intelligence proposes that EI is based on self-perceptions of one’s emotional abilities, which are grounded in an individual’s personality and can be assessed with self-report measures.

The Mayer-Salovey-Caruso Emotional Intelligence Test (MSCEIT) [28] was developed to measure EI-performance according to the four-branch model, as presented by Brackett and Salovey [29]. We have previously shown comparable overall EI (MSCEIT total score) in patients diagnosed with SZ or BD, albeit SZ patients displayed significantly more deficits in most MSCEIT branches [30]. The most distinct between-group difference was found in the “understanding emotions” section. In order to expand on this previous research, we investigate the relationship between EI and QoL in an extended sample of clinically stable outpatients, and a non-psychiatric control group, with a special focus on QoL measured on the basis of objective life circumstances.

### Study rationale and hypotheses

Social cognitive skills have been associated with better QoL in SMI [8, 11–13]. While previous research in this context mainly focused on emotion perception, the present study aimed to investigate the impact of the other subdomains of emotion processing on a subjective QoL estimate and objective QoL indicators. We hypothesized that patients with better performance in the MSCEIT report better QoL. Based on previous studies, we assumed that SZ and BD patients report comparable subjective QoL, whereas BD patients show higher levels of objective QoL.

## Methods

### Setting and sample

The study sample consisted of outpatients diagnosed with SZ or BD between 18 and 65 years of age. Recruitment and study assessments took place at the specialized outpatient clinics of the Department of Psychiatry, Psychotherapy and Psychosomatics of the Medical University Innsbruck and of the Department of Psychiatry and Psychotherapy of the Private Medical University Salzburg. Healthy controls matched for age and sex were recruited from the general community. All participants were native German speakers.

At the time of study inclusion, patients had to be clinically stable, without hospitalization for at least six months, and without any modifications in psychopharmacological treatment for three months. Exclusion criteria for all participants included: neurological and developmental disorders, and physical illnesses that might interfere with cognitive performance (unstable and/or severe organ system diseases, e.g. cardiovascular, endocrine, metabolic).

### Measures

#### Emotional intelligence

EI was assessed using the German pencil-and-paper version [31] of the MSCEIT [32, 33]. This instrument includes 141 items measuring the abilities of perceiving, using, understanding, and managing emotions. Similar to other intelligence tests, the average score is 100 with a standard deviation of 15. Higher scores indicate higher EI levels. The MSCEIT is both content and structurally valid (overall reliability r = 0.93), and shows discriminate validity from measures of analytic intelligence and many personality constructs [29].

#### Quality of life

QoL was assessed using the German version of the Lancashire Quality of Life Profile [34], the Berliner Lebensqualitätsprofil (BELP, [35]), which comprises 70 items and offers both a subjective QoL estimate and objective QoL indicators. For the subjective QoL estimate, the BELP focuses on the following nine domains: work/occupation, leisure time, financial situation, housing, personal safety, family life, friends, physical health, and mental health. Patients are asked to rate their satisfaction on a seven-point scale with a score of 1 indicating “poorest quality of life” and a score of 7 indicating “optimal quality of life”. Objective QoL indicators are assessed via several items enquiring about objective life circumstances, e.g., the number of leisure activities, or the number of days per week of contacts with friends. Following Ruggeri and coworkers [36], we calculated seven subscores: work/occupation (hours per week), number of leisure time activities, contacts with family members and friends (days per week), social contacts outside psychiatry (days per week), physical health problems (score calculated from the items “frequency of utilization of physical health services “ and “reduced mobility”), and mental health problems (score calculated from the items “frequency of utilization of mental health services “ and “hospitalization due to mental health problems”). The scales’ validity properties have been shown to be satisfactory (Cronbach’s α = 0.88 for overall QoL and 0.93 for all subscales together) [37].

### Procedure

Sociodemographic and clinical characteristics were collected via structured interviews: the Mini International Neuropsychiatric Interview (M.I.N.I., [38]) was used to confirm diagnoses in both patient groups, to assess a history of psychosis in BD patients, and to exclude any Axis I disorder (with the exception of nicotine dependence) according to DSM-IV in controls. Psychopathology was assessed by means of the Positive and Negative Syndrome Scale (PANSS, [39]) in SZ patients and using the German version [40] of the Young Mania Rating Scale (YMRS, [41]) and the Montgomery-Åsberg Depression Rating Scale (MADRS, [42]) in BD patients. A structured interview was used to assess family history for affective or other psychotic disorders in first-degree relatives of control subjects.

One to three individually timed sessions per participant were offered to complete all study procedures.

All procedures contributing to this work complied with the standards of the local ethics committees and were conducted according to Good Clinical Practice (GCP) standards on human experimentation and with the Helsinki Declaration of 1975, as revised in 2008. The study was approved by the local ethics committees and all participants provided written informed consent. Procedures were performed by a trained research team consisting of psychiatrists and master level clinical psychologists.

### Statistical Methods

Prior to the analysis, all metric variables were checked for deviations from normality by means of the Shapiro–Wilk test. Group comparisons with regard to sample characteristics were performed by one-way analysis of variance, Kruskal–Wallis test, and Chi-square test, depending on the variable type (normally distributed, non-normally distributed, and categorical, respectively). The Kruskal–Wallis test was also employed for comparing the three groups with respect to EI and QoL, as the MSCEIT total score and the majority of the BELP subscales were not normally distributed. Associations between EI and QoL were investigated using Spearman rank correlations, as the majority of the variables involved showed significant deviations from normality.

The combined effects of sociodemographics (age, sex, education) and EI (subscales of the MSCEIT) on objective QoL were investigated by hierarchical regression analysis. Linear regression was used for approximately normally distributed dependent variables and ordinal regression analysis for non-normally distributed or ordinal variables. First, the three sociodemographic variables were entered irrespective of their statistical significance to ensure that testing is adjusted for sociodemographics. Subsequently, the subscales of the MSCEIT were added to the regression model by means of forward stepwise variable selection. To quantify the effects of the independent variables, partial correlation coefficients were reported in the case of linear regression and odds ratios in the case of ordinal regression. To reduce the number of dependent variables, the three QoL indicators of the social domain were summarized to one social QoL score by adding up the Z-transformed values of the individual items.

## Results

### Study sample

The study sample consisted of 123 outpatients with SZ (*n* = 63) or BD (*n* = 60) and 80 healthy controls. Sociodemographic and clinical characteristics are shown in Table 1. The three groups were comparable with respect to age and sex, but differed in education and marital status. Controls had a significantly higher level of education than both patient groups, and the proportion of singles was significantly higher in SZ patients than in the two other groups.

**Table 1:** Sample characteristics

Variable	Category or unit	Group			Comparison	
		Schizophrenia N = 63	Bipolar Disorder N = 60	Healthy controls N = 80	Statistics ^a^	p-value
Age	Years	44.8 ± 10.1	46.3 ± 11.5	44.6 ± 10.3	F = 0.52	0.597
Sex	Male	37 (58.7%)	33 (55.0%)	45 (56.3%)	χ^2^ = 0.18	0.913
	Female	26 (41.3%)	27 (45.0%)	35 (43.8%)		
Education	Years	12.7 ± 3.1	13.1 ± 2.9	14.8 ± 3.3 ^b^	χ^2^ = 16.68	< 0.001
Duration of illness	Years	15.4 ± 10.4	14.2 ± 10.5	–	Z = 0.776 ^c^	0.438
BD with history of psychosis, N (%)		–	25 (43.1)	–		
Psychotropic Medication^d^						
Antipsychotics, N (%)	±	62 (98.4)	43 (71.7)	–		
Mood Stabilizer, N (%)		7 (11.1)	39 (65.0)	–		
Antidepressants, N (%)		18 (28.6)	25 (41.7)	–		
Benzodiazepines, N (%)		16 (25.4)	5 (8.3)	–		
PANSS total score, mean ± SD		53.9 ± 12.9	–	–	–	–
PANSS positive symptoms, mean ± SD		12.5 ± 5.1				
PANSS negative symptoms, mean ± SD		14.6 ± 4.9				
PANSS general symptoms, mean ± SD		26.7 ± 6.4				
YMRS, mean ± SD		-	3.33 ± 4.30	–	–	–
MADRS, mean ± SD		-	6.70 ± 6.29	–	–	–

*BD* Bipolar Disorder, *PANSS* Positive and Negative Syndrome Scale, *MADRS* Montgomery Asberg Depression Rating Scale, *YMRS* Young Mania Rating Scale

Values are shown as mean ± standard deviation or N (%)

^a^Always 2 degrees of freedom (d. f.) unless stated otherwise. Analysis by one-way analysis of variance (F), Kruskal–Wallis test (χ^2^), or Mann–Whitney U-test (Z)

^b^Significantly higher level of education in the control group than in the two patient groups (p < 0.01)

^c^d.f. = 2

^d^Mood stabilizier” = Lithium, Valproic acid, Lamotrigine and Topiramate; Second generation antipsychotics used as mood stabilizer are listed under “Antipsychotics”

### Emotional intelligence

SZ and BD patients had comparable MSCEIT total scores but scored significantly lower than healthy controls (see Table 2 for details). Looking at the branches separately revealed that in three out of four subscales (“using”, “understanding”, and “managing emotions”) SZ patients achieved significantly lower scores than those suffering from BD. Both patient groups scored significantly lower than controls in these three subscales, while no group difference emerged for the “perceiving emotions” branch.

**Table 2:** Emotional Intelligence and quality of life

Variable	SZ	BD	HC	Statistics^a^ (χ^2^)	p-value	Pairwise comparison
Emotional intelligence						
MSCEIT total score	88.0 ± 19.6	94.1 ± 19.1	105.6 ± 15.4	33.26	< 0.001	(SZ), (BD) < (HC)
MSCEIT Perceiving Emotions	100.5 ± 18.5	98.60 ± 16.9	103.3 ± 15.6	3.07	0.216	n.s
MSCEIT Using Emotions	95.9 ± 17.8	101.0 ± 18.0	106.7 ± 13. 0	14.71	< 0.001	(SZ) < (HC)
MSCEIT Understanding Emotions	79.1 ± 22.4	89.1 ± 18.9	99.7 ± 14.8	30.60	< 0.001	(SZ) < (BD) < (HC)
MSCEIT Managing Emotions	83.3 ± 18.3	92.6 ± 19.0	105.1 ± 13.8	45.17	< 0.001	(SZ) < (BD) < (HC)
Subjective QoL (range 1–7)						
Global QoL	4.86 ± 1.47	4.89 ± 1.29	5.84 ± 0.78	27.43	< 0.001	(SZ), (BD) < (HC)
Work/occupation	4.72 ± 1.63	4.70 ± 1.36	5.01 ± 1.20	1.71	0.424	n. s
Leisure time	5.01 ± 1.29	4.73 ± 1.24	5.71 ± 0.92	24.20	< 0.001	(SZ), (BD) < (HC)
Family life	5.14 ± 1.60	5.48 ± 1.17	5.87 ± 0.86	6.40	0.041	(SZ) < (HC)
Friends	5.15 ± 1.32	5.29 ± 1.28	6.30 ± 0.81	43.47	< 0.001	(SZ), (BD) < (HC)
Physical health	5.11 ± 1.56	5.24 ± 1.20	5.97 ± 1.06	19.07	< 0.001	(SZ), (BD) < (HC)
Mental health	4.82 ± 1.62	4.74 ± 1.48	6.35 ± 0.8	54.64	< 0.001	(SZ), (BD) < (HC)
Objective QoL						
Work/occupation (hours per week) Proportion without work (0 h)	8.48 ± 15.79 68.9%	16.97 ± 17.78 31.6%	33.56 ± 11.32 1.3%	67.52	< 0.001	(SZ) < (BD) < (HC)
Leisure time activities (0–6)	2.61 ± 0.98	2.55 ± 1.59	3.44 ± 0.65	29.56	< 0.001	(SZ), (BD) < (HC)
Contacts with family (frequency, score 0–5)	3.32 ± 1.41	3.37 ± 1.38	3.95 ± 0.81	9.53	0.009	(SZ), (BD) < (HC)
Contacts with friends (days per week)	3.24 ± 2.70	2.80 ± 2.08	3.51 ± 2.15	3.36	0.186	n.s
Social contacts outside psychiatry (days per week with person not related to psychiatry)	4.20 ± 2.96	5.04 ± 2.44	5.86 ± 2.07	11.58	0.003	(SZ), (BD) < (HC)
Physical health problems Number of physical illness items checked (0–2)	0.69 ± 0.78	0.80 ± 0.74	0.65 ± 0.75	1.82	0.402	n.s
Mental health problems Number of mental illness items checked (0–2)	1.32 ± 0.65	1.13 ± 0.81	0.02 ± 0.13	113.21	< 0.001	(SZ), (BD) > (HC)

*SZ* patients with schizophrenia, *BD* patients with bipolar-I-disorder, *HC* healthy control subjects, *QoL* quality of life, *MSCEIT* Mayer-Salovey-Caruso Emotional Intelligence Test

n.s. = not significant (i.e. no significant differences between the three groups)

The Berliner Lebensqualitätsprofil-items “financial situation”, “housing” and “personal safety” in the subjective QoL section were omitted for reasons of space

^a^Kruskal–Wallis test, giving rise to a χ^2^ value as the corresponding test statistic

### Quality of life

The two patient groups were comparable with regard to most subjective and objective QoL indicators, but reported significantly lower QoL compared to controls in most domains assessed by the BELP. Notably, the three groups were comparable in the areas of work/occupation (subjective QoL), as well as the frequency of both contacts with friends, and utilization of physical health services (objective QoL) (see Table 2 for details).

Both BD patients with and without a history of psychosis and control subjects with and without a family history for psychotic disorders did not differ with regard to EI (total MSCEIT and subscales) or QoL scores.

### Association between emotional intelligence and quality of life

In SZ patients, the MSCEIT total score correlated with only one subjective QoL indicator (safety, r = 0.29, p = 0.032) while no significant correlation emerged in patients suffering from BD. Uniquely in controls, several significant correlations between the MSCEIT total score and subjective QoL were found (family life: r = 0.26, p = 0.004; mental health: r = 0.27, p = 0.002; safety: r = 0.19, p = 0.036), which were supported by significant associations with the MSCEIT branches “perceiving emotions “ and “managing emotions “.

Correlations between EI and objective QoL indicators are shown in Table 3. In SZ patients, the MSCEIT total score showed a significant positive correlation with the BELP domains “contacts with family members” and “social contacts outside psychiatry”, and a significant negative correlation with “utilization of mental health services”. Of the MSCEIT branches, “managing emotions” exhibited most (positive) correlations with objective QoL indicators. Among the other two groups, EI and objective QoL indicators showed only very few significant associations: in BD patients, “managing emotions”, “using emotions”, and the MSCEIT total score correlated positively with the BELP domain “social contacts outside psychiatry”. In healthy controls, “managing emotions” correlated positively with “social contacts outside psychiatry”, whereas “using emotions” correlated negatively with “physical health problems”.

**Table 3:** Correlation between Emotional Intelligence (MSCEIT) and objective quality of life indicators (BELP) – Spearman rank correlation coefficients

Group	QoL domain	MSCEIT total score	Perceiving emotions	Using emotions	Understanding emotions	Managing emotions
Schizophrenia (n = 57-62^a^)	Work/occupation (hours per week)	.255	.005	.110	.240	.291^*^
	Leisure time activities (# of activities. 0–6)	.120	.001	.212	.085	.194
	Contacts with family (score 0–5)	.356^**^	.114	.249	.366^**^	.285^*^
	Contacts with friends (days per week)	.244	.159	.158	.165	.342^**^
	Social contacts outside psychiatry (days per week)	.330^*^	.257^*^	.208	.400^**^	.122
	Physical health problems (# items checked. 0–2)	− .056	.079	− .265^*^	− .178	− .032
	Mental health problems (# items checked. 0–2)	− .275^*^	− .158	− .483^**^	− .204	− .132
Bipolar disorder (n = 53–58 ^a^)	Work/occupation (hours per week)	− .062	.010	− .104	− .155	− .029
	Leisure time activities (# of activities. 0–6)	.102	.019	− .045	.138	.081
	Contacts with family (score 0–5)	.000	− .085	− .093	.234	.081
	Contacts with friends (days per week)	.193	.050	.152	.115	.170
	Social contacts outside psychiatry (days per week)	.314^*^	.111	.289^*^	.220	.293^*^
	Physical health problems (# items checked. 0–2)	.090	− .106	− .034	.176	.154
	Mental health problems (# items checked. 0–2)	.189	.164	.192	.132	.024
Control	Work/occupation (hours per week)	− .077	− .165	.029	− .111	− .038
	Leisure time activities (# of activities. 0–6)	− .023	− .031	− .063	− .014	.044
	Contacts with family (score 0–5)	.149	.110	.116	.001	.218
	Contacts with friends (days per week)	.006	.160	-.038	− .120	.181
	Social contacts outside psychiatry (days per week)	.226	.066	.179	.172	.335^**^
	Physical health problems (# items checked. 0–2)	− .189	− .078	-.303^**^	− .112	− .157
	Mental health problems (# items checked. 0–2)	− .114	− .102	− .111	− .061	.010

*QoL* quality of life, *BELP* Berliner Lebensqualitätsprofil, *MSCEIT* Mayer-Salovey-Caruso Emotional Intelligence Test

^a^Due to varying numbers of missing values per QoL domain

*p < 0.05, **p < 0.01

### Combined effect of sociodemographic variables and EI on objective QoL – results of regression analysis

Findings of the regression analysis are presented in Table 4. Regarding the effect of EI on objective QoL, the results of the regression analysis support findings of the correlation analyses in most cases. Only if two different MSCEIT branches correlated significantly with the same QoL domain, just one branch was retained in the regression model. Thus, in SZ patients the MSCEIT branch “managing emotions” showed a trend towards predicting objective QoL in the work domain (*p* = 0.054). Furthermore, “understanding emotions” significantly predicted QoL in the social domain (*p* = 0.019), and “using emotions” was associated with better QoL both in physical and in mental health (*p* = 0.035 and *p* < 0.001, respectively). Among sociodemographic variables, higher education levels (*p* = 0.044) and, at a trend level, female sex (p = 0.064) predicted higher scores of leisure time activities. No other QoL domain was significantly related to any of the sociodemographic variables tested.

**Table 4:** Results of regression analyses^a^

Group	Dependent variable (QoL domain)	Model 1: Sociodemographics only (all variables entered)			Model 2: Sociodemographics plus EI (significant variables added by forward selection)		Test for significant effect of EI (Model 2 vs Model 1)		
		Significant Predictors ^c^	Coefficients of significant predictors ^d^	R^2^ or Nagelkerke R^2^	Variable	Coeff. ^c^	Total R^2^	F or χ^2^ for change in R^2^	p-value
Schizophrenia	Work	–	–	R^2^ = 0.080	Managing E	β = 0.26	R^2^ = 0.143	F _1,53_ = 3.9	(0.054)
	Leisure	Sex f Edu	β = 0.24 β = 0.30	R^2^ = 0.147	–	–	R^2^ = 0.147	–	–
	Social contacts^b^		–	R^2^ = 0.111	Understanding E	β = 0.35	R^2^ = 0.200	F_1,53_ = 5.8	0.019
	PH problems	–	–	R^2^_Nag_ = 0.141	Using E	OR = 0.60	R^2^_Nag_ = 0.216	χ^2^ = 4.5	0.035
	MH problems	Edu	OR = 0.36	R^2^_Nag_ = 0.212	Using E	OR = 0.27	R^2^_Nag_ = 0.431	χ^2^ = 11.2	< 0.001
Bipolar disorder	Work	–	–	R^2^ = 0.100	–	–	R^2^ = 0.100	–	–
	Leisure	Age Edu	β = -0.29 β = 0.23	R^2^ = 0.212	–	–	R^2^ = 0.212	–	–
	Social contacts^b^	Edu	β = 0.41	R^2^ = 0.199	–	–	R^2^ = 0.199	–	–
	PH problems	Age	OR = 0.55	R^2^_Nag._ = 0.109	–	–	R^2^_Nag._ = 0.109	–	–
	MH problems	Edu	OR = 0.44	R^2^_Nag._ = 0.179	–	–	R^2^_Nag._ = 0.179	–	–
Control	Work	Sex m	β = 0.32	R^2^ = 0.111	–	–	R^2^ = 0.111	–	–
	Leisure	–	–	R^2^ = 0.013	–	–	R^2^ = 0.013	–	–
	Social contacts^b^	–	–	R^2^ = 0.026	Managing E	β = 0.44	R^2^ = 0.216	F_1,75_ = 17.1	< 0.001
	PH problems	–	–	R^2^_Nag._ = 0.013	Using E	OR = 0.64	R^2^_Nag._ = 0.106	χ^2^ = 5.9	0.015
	MH problems	–	–	R^2^_Nag._ = 0.025	–	–	R^2^_Nag._ = 0.025	**–**	–

*OR* odds ratio, *EI* Emotional Intelligence, *MSCEIT* Mayer-Salovey-Caruso Emotional Intelligence Test, *PH* physical health, *MH* mental health, *m* male, *f* female, *Edu* education, *Managing E*. Managing Emotions (likewise for other MSCEIT subscales), R^2^ = coefficient of determination, R^2^_Nag_ = Nagelkerke R^2^

^a^The variables work, leisure and social contacts were analyzed by multiple linear regression, physical health problems and mental health problems by ordinal regression

^b^Composite score combining contacts with family, contacts with friends, and social contacts outside psychiatry (see Statistical Methods)

^c^Only significant predictors shown (i.e., independent variables with p < 0.05 in the regression model 1)

^d^Standardized beta coefficient (β) or odds ratio (OR)

In BD patients, none of the EI branches were significantly associated with any of the QoL domains (which is in accordance with the correlation analyses, except for one domain, i.e. “social contacts”). Of the sociodemographic variables tested, higher age was associated with fewer leisure time activities and fewer physical health problems (p = 0.044 and p = 0.030, respectively). Higher education levels predicted higher scores in leisure time activities (p = 0.043) and social contacts (p = 0.003). Higher education level was also associated with fewer mental health problems (p = 0.002).

In healthy controls, “managing emotions” significantly predicted QoL in the social domain (p < 0.001); “using emotions” was associated with fewer physical health problems (p = 0.015). Male sex was associated with higher objective QoL in the work domain (p = 0.014).

## Discussion

This cross-sectional study investigated the relationship between the social cognitive domain of Emotional Intelligence and quality of life in patients suffering from schizophrenia or bipolar I disorder. Of note and contrary to previous research, both subjective and objective QoL indicators were considered, i.e., subjective satisfaction with specific areas of life, and objective life circumstances (e.g., hours of occupation per week or frequency of contacts with family members and friends). The two patient groups showed comparable levels of overall EI and were largely comparable with regard to both subjective and objective QoL. However, the patient groups indicated significantly lower levels of EI and QoL compared to healthy controls. A significant relationship between EI and objective QoL was particularly seen in schizophrenia patients, however, overall effect sizes were small.

Our sample consisted of chronically ill outpatients with an average duration of illness of about 15 years, and mostly mild symptoms, who had been stable both from a symptomatic and a medication perspective before study inclusion. We therefore were able to study the persistent impairments associated with SMI, rather than the transient changes associated with episodes of relapse. In line with previous findings from our group [30], SZ and BD patients had comparable overall EI (MSCEIT total score) but differed significantly in most MSCEIT branches, with BD patients displaying less severe deficits. Although the MSCEIT total score lay within general population norms in both patient groups, healthy controls outperformed both patient groups.

Expectedly, and in line with earlier investigations [13, 43, 44], patients indicated lower QoL compared to controls, with no significant difference between patient groups. Nonetheless, our findings re-emphasize that both SZ and BD patients are in need of continuous psychosocial care, even when clinically stable and merely mildly ill.

With regard to a potential association between social cognitive abilities and QoL in SZ, a positive correlation between ToM capabilities and QoL has been reported [45]. Interestingly, that study did not find any association between emotion perception abilities and QoL, whereas others reported a positive correlation between the ability to perceive emotions and family network relationships [46]. Among the present schizophrenia sample, patients achieving higher scores in the “perceiving emotions”, “managing emotions” (i.e., emotion regulation), and “understanding emotions” branches of the MSCEIT, reported a higher frequency of social contacts. Intuitively, this makes sense, as impairments in these areas likely lead to difficulties in building and maintaining social relationships. It should be noted, however, that the two mentioned studies, and the present investigation, differ notably with regard to age and duration of illness of participants, and used different instruments to investigate social cognitive functioning. Accordingly, the three samples are not entirely comparable. Nevertheless, individuals with schizophrenia often feel socially isolated and disconnected from friends and family, which is partly due to lacking social emotional abilities [47].

In BD patients, “frequency of social contacts outside psychiatry” was the only BELP domain that positively correlated with EI. In contrast, Aparicio et al. did not find any significant correlation between EI and interpersonal relationships [48]. Contrary to our investigation, their study had exclusively included euthymic patients and did not use a specific instrument to measure QoL. However, mean MADRS and YMRS scores were also very low in the present BD sample and further studies are needed to investigate this issue.

Recently, the relationship between the “managing emotions” branch of the MSCEIT and psychosocial functioning in SMI was examined. Significant positive associations in SZ, but not in BD (except an association between “managing emotions” and “interpersonal relations”), were reported [49], which is in line with our results. We suggest that the observed differences between diagnostic groups may be caused by differences in illness severity, since BD patients were symptomatically remitted, as indicated by very low MADRS and YMRS mean scores, whereas schizophrenia patients cannot be regarded as remitted, even though a PANSS mean score of 53.9 ± 12.9 indicates merely mild symptom severity. Since ToM and EI as assessed with the MSCEIT inter-related and are partly overlapping constructs [50], one can further speculate on the relevance of ToM, i.e. the ability to understand the mental states of others and “to reflect one`s own and others minds” [51] in this regard. ToM is a known prerequisite for empathy and is thus essential for social interactions in everyday life. Both, patients with SZ and BD have previously been shown to have marked ToM impairments [52] which in SZ, have been associated with reduced QoL [45]. Differences in ToM performance may therefore be the underlying factor for the stronger relationship between EI and QoL in our schizophrenia sample, however, further studies are needed to confirm this assumption.

According to our findings, patients suffering from SZ or BD may have different needs to achieve recovery, and a positive effect of a training of socioemotional abilities on patients’ QoL, may be expected in those suffering from SZ. Previous studies point to positive long-term effects of an EI training program on clinical outcomes in SZ patients [53]. From a clinical perspective, it would be of interest to investigate to what extent such a training also impacts QoL and whether this effect might persist over time.

Our study has several limitations. Firstly, a larger sample might have revealed more and stronger associations between the different subdomains of EI and QoL, and clearly, the cross-sectional design does not allow conclusions on causality. Secondly, we disregarded the potential influence of medication on the outcomes studied. Clearly, the compounds prescribed to our patients may have had a different impact on outcomes. However, as all patients were clinically stable, we can at least disregard efficacy differences between the different drugs. Lastly, it is debatable whether the objective QoL indicators calculated in this study suffice as “objective” measures without a third-party verification.

In summary, our results show that EI and QoL are interrelated. As hypothesized, this association was more pronounced in SZ than in BD patients, however, overall effect sizes were small. Accordingly, our findings should be interpreted cautiously, and first and foremost need to be replicated in larger samples. In a further step, it should be investigated whether social cognitive training programs targeting EI may especially help SZ patients to promote their QoL.

## Data Availability

Research data are not shared due to privacy concerns.
